# IR808-ATIPA: A Dual-Function Agent for Enhanced Computed Tomography Imaging and Radiotherapy Sensitization in Cervical Cancer Treatment

**DOI:** 10.34133/bmr.0222

**Published:** 2025-08-18

**Authors:** Kejin Liu, Rourou Zuo, Zhe Wang, Guoliang Chen, Xuefei Bao, Hongbo Wang, Hongzan Sun

**Affiliations:** ^1^ Liaoning Provincial Key Laboratory of Medical Imaging, Shenyang, China.; ^2^ Department of Nuclear Medicine, Shengjing Hospital of China Medical University, Shenyang, China.; ^3^ Key Laboratory of Structure-Based Drug Design & Discovery, Ministry of Education, Shenyang Pharmaceutical University, Shenyang, China.; ^4^ Department of Interventional Therapy, Shengjing Hospital of China Medical University, Shenyang, China.

## Abstract

Radiotherapy is pivotal in localized cancer treatment, yet balancing therapeutic efficacy with collateral tissue damage remains challenging. Conventional iodinated contrast agents, limited by rapid metabolism and short imaging windows, hinder precise radiotherapy planning. We developed IR808-ATIPA, a tumor microenvironment-responsive iodine-based compound integrating computed tomography (CT) imaging and radiosensitization. Synthesized by covalently linking IR808 and ATIPA, IR808-ATIPA leverages iodine’s x-ray attenuation for high-contrast imaging while enhancing radiation dose deposition in cervical cancer. Unlike conventional agents, its prolonged tumor retention improves imaging accuracy and therapeutic targeting. Evaluations in HeLa tumor-bearing nude mice demonstrated superior in vitro/in vivo imaging performance and sustained tumor accumulation. RNA sequencing revealed that IR808-ATIPA enhances radiotherapy efficacy by activating the ferroptosis pathway via increased reactive oxygen species production and amplified x-ray absorption. Safety assessments confirmed no notable toxicity to major organs. IR808-ATIPA functions dually as a CT contrast agent for precise tumor delineation and a radiosensitizer promoting ferroptosis-mediated radiotherapy enhancement. Its extended intratumoral retention enables targeted therapy, minimizing off-target effects. These findings highlight IR808-ATIPA as a promising theranostic agent, bridging imaging-guided precision and therapeutic efficacy to advance personalized cancer treatment.

## Introduction

Radiotherapy is one of the primary treatment modalities for various cancers in clinical settings, employing high-energy x-rays to destroy cancer cells through direct and indirect mechanisms. Direct action results from the interaction of ionizing radiation with biomolecules without intermediaries, mainly affecting single- and double-stranded DNA [1,2]. Indirect action involves the ionization of water molecules in tissues, generating hydroxyl radicals and other reactive oxygen species (ROS), which can cause damage to biomolecules and DNA [3–5]. However, the efficacy and dosage of radiotherapy are constrained by toxicity to normal tissues, as excessive irradiation can lead to severe side effects [6–8].

In clinical radiotherapy, localization through computed tomography (CT) imaging is an essential step. The real-time monitoring capabilities of imaging can effectively prevent both undertreatment and overtreatment. Due to its wide availability, efficiency, relatively low cost, and good tissue penetration, CT is extensively used in clinical practice [9–11]. Image-guided radiotherapy (IGRT) significantly enhances treatment outcomes and patient prognosis [12,13]. Accurate delineation and localization of the target area before radiotherapy are key steps for precision radiotherapy. Imaging-assisted target delineation allows for precise identification of the tumor’s location, shape, and extent, ensuring that radiation doses are focused on the tumor region, avoiding underdosing due to unclear boundaries, thereby reducing the risk of local recurrence [14–16]. Accurate target delineation also optimizes dose distribution, minimizing radiation damage to surrounding healthy tissues [17,18]. Combining imaging for precise delineation enables real-time monitoring of tumor position changes during treatment, allowing for timely adjustments to the radiotherapy plan.

Radiosensitization is the process of increasing the sensitivity of cancer cells to radiation damage. Radiosensitizers can store radiation energy and generate free radicals, thereby enhancing the radiosensitivity of tumor cells [19,20]. These agents can concentrate radiation energy within the tumor tissue or reduce the tumor’s resistance to x-rays, improving treatment efficacy without increasing radiation dosage and potentially expanding the therapeutic window [21]. Currently, radiosensitizers based on high atomic number (*Z*) materials have been utilized in cancer treatment [22,23]. These materials offer chemical stability, slow metabolism, high selectivity, and significant effects at low doses [22,24,25]. However, the biological safety and long metabolic cycles of metal materials remain significant drawbacks.

Iodine, as a nonmetallic high-*Z* material, possesses excellent x-ray absorption capabilities and biocompatibility, making it a commonly used contrast agent in clinical CT imaging and showing promise in enhancing radiotherapy [26,27]. While iodine agents theoretically have the potential for integrated diagnosis and therapy, there are challenges with existing clinical contrast agents: Their small molecular size results in insufficient retention time within tumors, not covering the therapeutic window of radiotherapy plans [28]; moreover, traditional iodine contrast agents only enhance radiation absorption without incorporating bioactive modules, failing to achieve synergistic enhancement of radiosensitization and tumor microenvironment modulation.

To address these challenges, we propose the design and synthesis of a novel iodine-containing compound, IR808-ATIPA, through covalent conjugation of IR808 with 5-amino-2,4,6-triiodoisophthalic acid (ATIPA). IR808-ATIPA is hypothesized to increase intracellular iron accumulation, a critical factor in ferroptosis induction, through mechanisms involving enhanced ROS generation, fatty acid metabolism, and lipid peroxidation (LPO). Combining IR808-ATIPA with x-ray irradiation is expected to simultaneously activate both ferroptosis and apoptosis pathways in tumor cells. This multimodal cell death approach aims to overcome the limitations associated with apoptosis, particularly the propensity for tumor cells to evade apoptotic pathways.

The rational design of IR808-ATIPA seeks to extend tumor retention by increasing molecular size, thus providing prolonged CT imaging capabilities that align with the entire radiotherapy process. This extended imaging window facilitates precise delineation of tumor target areas and enables personalized radiotherapy planning. Additionally, by simultaneously inducing ferroptosis and apoptosis, IR808-ATIPA enhances radiotherapy efficacy, providing a comprehensive strategy to improve therapeutic outcomes.

## Materials and Methods

### Synthesis of IR808-ATIPA

#### 2,3,3-Trimethyl-3*H*-indol-1-ium

Phenylhydrazine (5.00 g, 46.24 mmol) was dissolved in glacial acetic acid (100.00 ml) at ambient temperature. To this solution was added 3-methylbutan-2-one (5.97 g, 69.31 mmol, 1.5 equiv) under vigorous stirring. The reaction mixture was heated under reflux at 118 °C using an oil bath for 8 h. Upon completion [monitored by thin-layer chromatography (TLC)], the system was cooled to room temperature and concentrated in vacuo to afford a yellow viscous residue. The crude product was extracted by dichloromethane (50 ml) and saturated sodium bicarbonate solution (50 ml × 3). The combined organic layers were dried over anhydrous Na₂SO₄ (10 g) for 12 h, filtered through a sintered funnel, and concentrated under reduced pressure to yield the title compound as a dark red viscous oil (6.64 g, 90.21%).

#### 1-(5-Carboxypentyl)-2,3,3-trimethyl-3*H*-indolium bromide

Intermediate 1 (6.64 g, 41.62 mmol) and 6-bromohexanoic acid (16.21 g, 83.10 mmol, 2.0 equiv) were combined in a 100-ml round-bottom flask equipped with a reflux condenser. Anhydrous acetonitrile (60 ml) was added, and the mixture was heated to reflux (82 °C) under nitrogen atmosphere for 72 h using a temperature-controlled heating mantle. Reaction progress was monitored by TLC. Upon completion, the solvent was removed under reduced pressure to yield a viscous residue. The crude product was suspended in ethyl acetate (100 ml) and vigorously stirred at 0 °C for 2 h to induce precipitation. The resulting pink solid was collected by vacuum filtration through a Büchner funnel, washed with cold ethyl acetate (3 × 20 ml), and dried under high vacuum (0.5 mbar, 25 °C) for 12 h to afford the title compound as a crystalline pink powder (8.54 g, 58.23%).

#### 1-(6-((3,5-Dicarboxy-2,4,6-triiodophenyl)amino)-6-oxohexyl)-2,3,3-trimethyl-3*H*-indol-1-ium bromide

A solution of 1-(5-carboxypentyl)-2,3,3-trimethyl-3*H*-indolium bromide (0.50 g, 1.41 mmol) in anhydrous dichloromethane (10 ml) containing one drop of *N*,*N*-dimethylformamide (DMF) as catalyst was treated with oxalyl chloride (0.89 g, 7.05 mmol, 5.0 equiv) under argon atmosphere. The reaction mixture was stirred for 12 h at ambient temperature (25 °C) in a light-protected flask. After complete conversion was confirmed by TLC analysis, the solvent and excess reagent were removed under reduced pressure using a rotary evaporator. The resulting acyl chloride intermediate was immediately dissolved in *N*,*N*-dimethylacetamide (DMAc; 10 ml) and added dropwise over 15 min to a vigorously stirred solution of ATIPA (0.79 g, 1.41 mmol) and triethylamine (0.43 g, 4.23 mmol, 3.0 equiv) in DMAc (10 ml) maintained at 0 to 5 °C. The reaction system was gradually warmed to 80 °C and maintained at this temperature for 5 h under continuous argon flow with light exclusion. Upon cooling to room temperature, the mixture was quenched by slow addition to ice-cold deionized water (100 ml), inducing precipitation of the crude product. The solid was collected by vacuum filtration, washed with cold water (3 × 20 ml), and dried under high vacuum. Final purification was achieved by flash chromatography on silica gel using a gradient elution of dichloromethane/methanol (10:1 v/v), yielding the desired compound as a white crystalline powder (0.15 g, 12.1% yield).

#### *N*-((E)-(2-chloro-3-((E)-(phenylimino)methyl)cyclohex-2-en-1-ylidene)methyl)aniline hydrochloride

A 250-ml 3-necked flask equipped with a thermometer, reflux condenser, and drying tube was charged with anhydrous DMF (20 ml) and anhydrous dichloromethane (20 ml) under nitrogen atmosphere. The mixture was cooled to 0 °C using an ice-salt bath (−10 °C bath temperature) with vigorous stirring. A solution of phosphorus oxychloride (15 ml, 164.3 mmol, 3.2 equiv) in anhydrous dichloromethane (18 ml) was transferred to a pressure-equalizing dropping funnel. This solution was added dropwise (0.5 ml/min) to the reaction vessel over 1 h while maintaining the internal temperature below 5 °C. After complete addition, the mixture was stirred for 30 min at 0 °C before cyclohexanone (5.00 g, 50.94 mmol) was introduced via syringe pump over 15 min. The cooling bath was removed, and the reaction was heated to reflux (45 °C) for 3 h using a thermostated oil bath. The resulting dark solution was carefully quenched by pouring onto crushed ice (300 g) with mechanical stirring, followed by 12-h aging at 4 °C. The precipitated yellow solid was collected by vacuum filtration (Whatman No.4 filter paper), yielding crude product (4.12 g). The intermediate was dissolved in absolute ethanol (50 ml) and treated with aniline (6.69 g, 71.85 mmol, 1.4 equiv) at ambient temperature for 1 h. The reaction mixture was poured into 1 l of chilled 10% HCl (w/w) and stored at −20 °C for crystallization. The dark purple crystals were filtered through a sintered glass funnel (G4), dissolved in hot methanol (80 ml), and recrystallized by slow diffusion of methyl tert-butyl ether/n-hexane (1:1 v/v, 200 ml) to afford the final product as lustrous purple crystals (4.32 g, 23.67%).

#### 2-((E)-2-((E)-2-Chloro-3-(2-((E)-1-(6-((3,5-dicarboxy-2,4,6-triiodophenyl)amino)-6-oxohexyl)-3,3-dimethylindolin-2-ylidene)ethylidene)cyclohex-1-en-1-yl)vinyl)-1-(6-((3,5-dicarboxy-2,4,6-triiodophenyl)amino)-6-oxohexyl)-3,3-dimethyl-3*H*-indol-1-ium bromide

A 100-ml round-bottom flask equipped with a reflux condenser was charged with 1-(6-((3,5-dicarboxy-2,4,6-triiodophenyl)amino)-6-oxohexyl)-2,3,3-trimethyl-3*H*-indol-1-ium bromide (0.7600 g, 0.8083 mmol), *N*-((E)-(2-chloro-3-((E)-(phenylimino)methyl)cyclohex-2-en-1-ylidene)methyl)aniline hydrochloride (0.1300 g, 0.3800 mmol, 0.47 equiv), and anhydrous sodium acetate (0.0200 g, 0.244 mmol, 0.30 equiv) in absolute ethanol (15 ml). The system was purged with argon for 15 min and wrapped in aluminum foil for light-sensitive conditions. The mixture was heated under reflux for 8 h. Reaction progress was monitored by analytical TLC. Upon completion, the solvent was removed under reduced pressure to yield a dark green residue. The crude product was purified by flash chromatography on silica gel using a gradient elution of dichloromethane/methanol (50:1 to 20:1 v/v). Fractions containing the target compound were combined and concentrated in vacuo. The resulting material was further dried under high vacuum for 6 h to afford the title compound as a hygroscopic green crystalline powder (60.0 mg, 8.55%).

### Cell culture

HeLa cells (iCell-h088, authenticated via short tandem repeat analysis) were purchased from iCell Bioscience Inc. (Shanghai, China). Cells were cultured in Dulbecco’s modified Eagle’s medium (DMEM; Thermo Fisher Scientific) supplemented with 10% fetal bovine serum (FBS; Thermo Fisher Scientific) and 1% penicillin–streptomycin (Thermo Fisher Scientific) at 37 °C under a humidified atmosphere containing 5% CO₂.

### CCK-8 cell viability assays

Cell viability was assessed using the Cell Counting Kit-8 (CCK-8) assay. HeLa cells (5 × 10^3^ cells/well) were seeded into 96-well plates and cultured in DMEM (150 μl) for 24 h. Medium was replaced with fresh DMEM (150 μl) containing various concentrations of IR808-ATIPA (0 to 0.6 mg/ml). After 4-h incubation, cells were exposed to x-ray irradiation (0 or 4 Gy) and incubated for an additional 48 h. Subsequently, cells were washed with phosphate-buffered saline (PBS), and fresh DMEM (90 μl) containing 10 μl of CCK-8 reagent was added to each well. Absorbance was measured at 450 nm using a microplate reader after 2-h incubation.

### Clonogenic assays

HeLa cells (1 × 10^3^ cells/well) were seeded into 6-well plates and treated with either PBS or IR808-ATIPA for 4 h before exposure to x-ray irradiation (0 or 4 Gy). Cells were cultured for approximately 1 week, fixed with 4% paraformaldehyde for 30 min at room temperature, and stained with crystal violet solution for 15 min. Plates were gently washed with water and air-dried, and colonies were photographed. Untreated cells served as controls.

### Apoptosis detection

HeLa cells cultured in T25 flasks were divided into 4 groups: PBS, IR808-ATIPA, x-ray, and IR808-ATIPA plus x-ray. Cells were treated with PBS or IR808-ATIPA for 4 h, irradiated (0 or 4 Gy), and cultured for an additional 48 h. Cells and supernatants were collected, resuspended in 1× binding buffer, stained with Annexin V–fluorescein isothiocyanate (FITC) and propidium iodide (PI), and incubated at room temperature in the dark for 30 min. Apoptotic cells were quantified via flow cytometry. Apoptosis rate was calculated as follows:

Apoptosis rate (%) = (Early apoptotic cells + Late apoptotic cells)/Total cells × 100%.

### RNA sequencing

RNA sequencing was performed on the Illumina Novaseq X Plus platform with 3 biological replicates per group. Raw reads were processed using Fastp (version 0.18.0) for quality control, followed by alignment to the human genome via HISAT2 v2.1.0. The mapped reads of each sample were assembled by using StringTie v1.3.1 in a reference-based approach. For each transcription region, a FPKM (fragment per kilobase of transcript per million mapped reads) value was calculated to quantify its expression abundance and variations, using RSEM software. Differential expression analysis was performed with DESeq2 (false discovery rate-adjusted *P* < 0.05). Pathway enrichment analysis utilized the Kyoto Encyclopedia of Genes and Genomes (KEGG) database.

### Cell morphology and lipid droplet staining

HeLa cells were seeded in 96-well plates, treated with PBS or IR808-ATIPA for 4 h, irradiated (0 or 4 Gy), and incubated for 48 h. Cells were fixed with 4% paraformaldehyde for 10 to 15 min, washed with PBS, and stained using a solution containing BODIPY 493/503 (1:1,000), Hoechst 33342 (1:1,000), and assay buffer (1:1:998). After incubation for 10 to 20 min in the dark, cells were washed twice with PBS and observed under a fluorescence microscope.

### γH2AX immunofluorescence staining

Cells were cultured in 96-well plates, treated with PBS or IR808-ATIPA for 4 h, irradiated (0 or 4 Gy), and incubated for an additional 6 h. After fixation and washing, cells were blocked with immunostaining blocking solution and incubated overnight at 4 °C with anti-γH2AX primary antibody. Cells were washed and incubated with secondary antibody for 1 h at room temperature, followed by 4′,6-diamidino-2-phenylindole (DAPI) staining for 5 min. Cells were imaged using fluorescence microscopy (γH2AX: green fluorescence; nuclei: blue fluorescence).

### Intracellular ROS detection

Cells were cultured in 96-well plates, treated with PBS or IR808-ATIPA for 4 h, irradiated (0 or 4 Gy), and incubated for 48 h. Intracellular ROS were detected by staining with 2′,7′-dichlorodihydrofluorescein diacetate (DCFH-DA) (37 °C, 20 min). Cells were washed twice with PBS and analyzed under a fluorescence microscope. Untreated cells served as controls.

### Western blotting

HeLa cells were cultured in T25 culture flasks and treated with PBS or IR808-ATIPA in a CO₂ incubator for 4 h. After replacing the medium, cells were exposed to radiation (0 or 4 Gy) and further cultured for 48 h. Cells were then washed with PBS, and 100 μl of PBS was added to each flask. Cell suspensions were collected using a cell scraper and centrifuged at 4 °C (3,000 rpm for 5 min). The supernatant was discarded, and the cell pellet was resuspended in radioimmunoprecipitation assay lysis buffer containing phenylmethylsulfonyl fluoride solution (99:1). The suspension was placed on ice and shaken for 30 min. Afterward, the samples were centrifuged again at 4 °C (12,000 rcf for 30 min), and the supernatant was retained. Protein concentrations were determined using a bicinchoninic acid (BCA) protein assay kit.

Equal amounts of protein (20 μg) were separated by sodium dodecyl sulfate–polyacrylamide gel electrophoresis (SDS-PAGE) and transferred to a polyvinylidene fluoride (PVDF) membrane. The membrane was blocked with a blocking solution for 20 min and incubated overnight with primary antibodies at 4 °C. The following primary antibodies were used: rabbit anti-ACSL4 (1:10,000, Abcam, catalog no. ab155282), rabbit anti-GPX4 (1:1,000, Abcam, catalog no. ab125066), and rabbit anti-β-tubulin (1:1,000, Cell Signaling Technology, catalog no. 2128).

The membrane was washed 3 times with tris-buffered saline Tween (TBST) (10 min each) and incubated with horseradish peroxidase-conjugated goat anti-rabbit immunoglobulin G (H+L) secondary antibody (1:10,000, Proteintech, catalog no. SA00001-2) at room temperature for 1 h. Chemiluminescent detection reagents were used to visualize the protein bands.

### Detection of intracellular oxidative stress biomarkers

HeLa cells cultured in T25 culture flasks were treated with PBS or IR808-ATIPA for 4 h. After changing the medium, cells were irradiated (0 or 4 Gy) and cultured for an additional 48 h. The cells were washed with PBS, and the levels of glutathione (GSH), glutathione peroxidase (GSH-PX), and malondialdehyde (MDA) were measured according to the manufacturer’s instructions. The values were normalized to the total protein mass using a BCA protein assay kit. Untreated cells served as the control group.

### Xenograft model

Five-week-old female BALB/c mice were purchased from Beijing Huafukang Bioscience Co. Ltd. Mice were randomly assigned to 4 groups (*n* = 6 per group). Each mouse was subcutaneously injected in the axillary region with a suspension containing 1 × 10^7^ HeLa cervical cancer cells in 100 μl of DMEM medium. Animals were housed under a standard 12-h light/dark cycle at controlled room temperature (20 to 23 °C). The sample size was calculated using G*Power 3.1 software (effect size *d* = 1.8, α = 0.05, β = 0.2), in compliance with the ARRIVE 2.0 guidelines.

The animal experiments were approved by the Ethics Committee of Shengjing Hospital, China Medical University (Ethics No.: 2024PS185K).

### In vitro CT imaging

CT values of IR808-ATIPA at various concentrations were assessed using a micro-CT system designed for small animals. Different concentrations of IR808-ATIPA solutions were aliquoted into microcentrifuge tubes and scanned using micro-CT. CT values were recorded to construct a curve representing the relationship between IR808-ATIPA concentration and CT intensity.

Micro-CT (for both in vitro and in vivo studies) parameters included the following: circular scan frames, 720; tube voltage, 70 kV; tube current, 550 μA; maximum field of view, 110 mm; distance source—detector, 622.5 mm; distance source—object, 240 mm; rebinning mode, 1 × 1; frame rate, 20 frames/s.

### In vivo CT imaging

After subcutaneous tumor formation in mice, 50 μl of IR808-ATIPA was injected into the tumor. CT imaging was performed at various time points: pre-injection and 3 min, 30 min, 1 h, 2 h, 3 h, and 4 h post-injection. ROI (region of interest) analysis was conducted to track changes in the CT values of the tumor over time.

### In vivo antitumor experiments

When tumors reached approximately 100 mm^3^, mice were randomly divided into 4 groups: group 1, receiving an intratumoral injection of PBS (50 μl) on day 0; group 2, receiving an intratumoral injection of IR808-ATIPA (50 μl) on day 0; group 3, receiving an intratumoral injection of PBS (50 μl) with x-ray irradiation (4 Gy) on day 0; group 4, receiving an intratumoral injection of IR808-ATIPA (50 μl) with x-ray irradiation (4 Gy) on day 0.

The in vivo experiment was terminated when any tumor dimension reached 15 mm, at which point all mice were euthanized. Tumor tissues and major organs were harvested and fixed in paraformaldehyde (4 wt %). Immunohistochemical staining for Ki67 and GPX4 was performed on tumor tissue sections.

Euthanasia was performed via CO_2_ inhalation at a flow rate of 30% chamber volume/min, followed by cervical dislocation to ensure death.

### Tissue pathological assessment

After completing the treatment regimen, mice were euthanized under anesthesia. Major organs (heart, liver, spleen, kidney, and lung) were collected using standardized dissection procedures. Tissues were fixed, sectioned, and stained with hematoxylin and eosin (H&E) for pathological analysis.

### Biosafety test and blood analysis

To assess the in vivo toxicity of IR808-ATIPA, healthy mice were injected with either PBS or IR808-ATIPA. Fifteen days post-injection, the mice were euthanized, and 1 ml of blood was collected from the heart into tubes containing 20 mg/ml EDTA. After centrifugation at 1,500 rpm for 10 min, serum was collected and analyzed for various biochemical markers, including blood urea (UREA), plasma uric acid (UA), aspartate aminotransferase (AST), alanine aminotransferase (ALT), and myocardial enzyme spectrum (CK).

### Statistical analysis

Data are expressed as mean ± SD, representing 3 independent biological replicates (*n* = 3). Normal distribution and homogeneity of variance were verified using Shapiro–Wilk (*P* > 0.05) and Levene’s tests (*P* > 0.1), respectively. Statistical analysis was performed using GraphPad Prism 9.5.1 software (GraphPad Software, USA). Two-way analysis of variance (ANOVA) was used to evaluate main effects and interactions between drug treatment and radiotherapy, followed by Tukey’s multiple comparisons test for intergroup differences. Statistical significance was defined as follows: ****P < 0.0001; ***P < 0.001; **P < 0.01; *P < 0.05; ns, not significant (*P* > 0.05).

## Results

### Synthesis and characterization of IR808-ATIPA

**Fig. 1.**
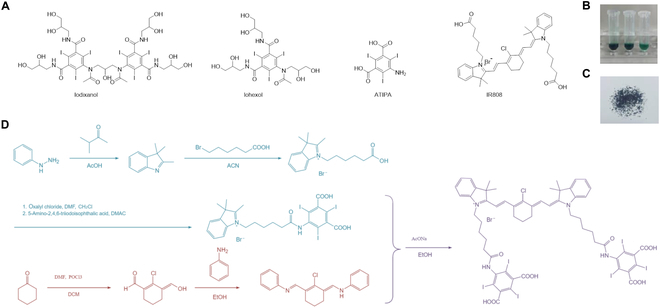
Synthesis and characterization of IR808-ATIPA. (A) Chemical structure of iohexol, iodixanol, IR808, and ATIPA. (B) Photographs of IR808-ATIPA solutions at varying concentrations. (C) Physical properties of IR808-ATIPA in gram scale. (D) Synthetic route for IR808-ATIPA.

Clinically used iodine-based contrast agents, such as iohexol and iodixanol, possess high iodine content, with their core component being ATIPA (Fig. 1A). This enables them to effectively absorb x-rays, significantly improving imaging quality [29]. Additionally, the high atomic number of iodine enhances radiation absorption, providing inherent radiosensitizing properties [30]. Therefore, we developed a novel radiosensitizer, IR808-ATIPA (Fig. 1B and C), by covalently linking IR808 with ATIPA (Fig. 1D), yielding a molecule with a high iodine content for enhanced imaging and radiosensitizing properties. The compound was characterized using nuclear magnetic resonance and high-resolution mass spectrometry, confirming a molecular weight of approximately 1,764.8 with an iodine content of about 43% (Fig. S1A to C and Tables S1 and S2).

### Cellular damage effects of IR808-ATIPA

**Fig. 2.**
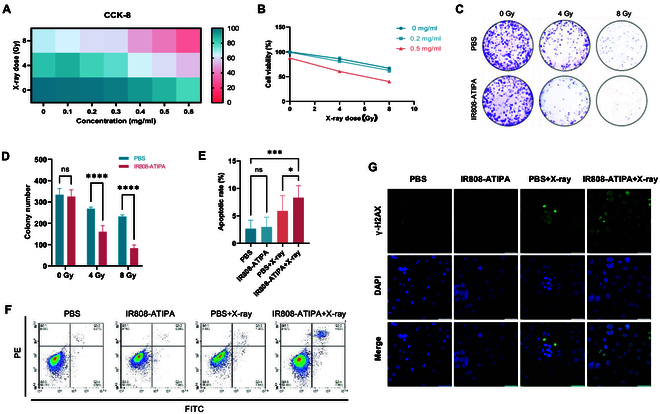
In vitro cellular damage assessment of IR808-ATIPA. (A and B) CCK-8 cytotoxicity assay results. (C and D) Colony formation assay results. (E and F) Flow cytometric analysis of cell apoptosis. (G) Immunofluorescence staining for γH2AX, indicating DNA damage (scale bar, 200 μm). Data are presented as mean ± SD (*n* = 3) and were analyzed by 2-way ANOVA with Bonferroni’s multiple comparison test. Significance levels: ****P < 0.0001; ***P < 0.001; **P < 0.01; *P < 0.05; ns, not significant (*P* > 0.05).

IR808-ATIPA enhances the uptake of x-rays by tumor cells, thereby amplifying cellular damage. Cytotoxicity assays revealed that the extent of cellular damage was dependent on both the concentration of IR808-ATIPA and the radiation dose (Fig. 2A and B). Specifically, IR808-ATIPA at a concentration of 0.5 mg/ml exhibited low intrinsic cytotoxicity but significantly sensitized cells to radiation therapy. In the absence of radiation, IR808-ATIPA alone caused minimal reduction in cell viability, indicating negligible intrinsic toxicity. However, under irradiation, cell viability decreased progressively with increasing IR808-ATIPA concentration. These findings were further corroborated by colony formation assays, where IR808-ATIPA demonstrated a substantially stronger inhibitory effect on colony formation in HeLa cells compared to cells treated with radiation alone (Fig. 2C and D).

Radiation-induced cellular damage is primarily characterized by apoptosis resulting from DNA damage. Compared to control groups, cells receiving the combined treatment of IR808-ATIPA and x-rays exhibited slightly elevated levels of early apoptosis, late apoptosis, and total apoptosis (Fig. 2E and F). Furthermore, the extent of DNA damage observed in the IR808-ATIPA plus x-ray group was greater than that seen with either IR808-ATIPA or radiation alone, suggesting that IR808-ATIPA potentially augments radiation-induced DNA damage (Fig. 2G).

### Transcriptomic analysis of IR808-ATIPA-treated HeLa cells

To explore the molecular mechanisms underlying IR808-ATIPA-mediated radiosensitization, RNA sequencing was performed on HeLa cells subjected to various treatments: PBS alone, IR808-ATIPA alone, PBS plus x-rays, and IR808-ATIPA plus x-rays.

**Fig. 3.**
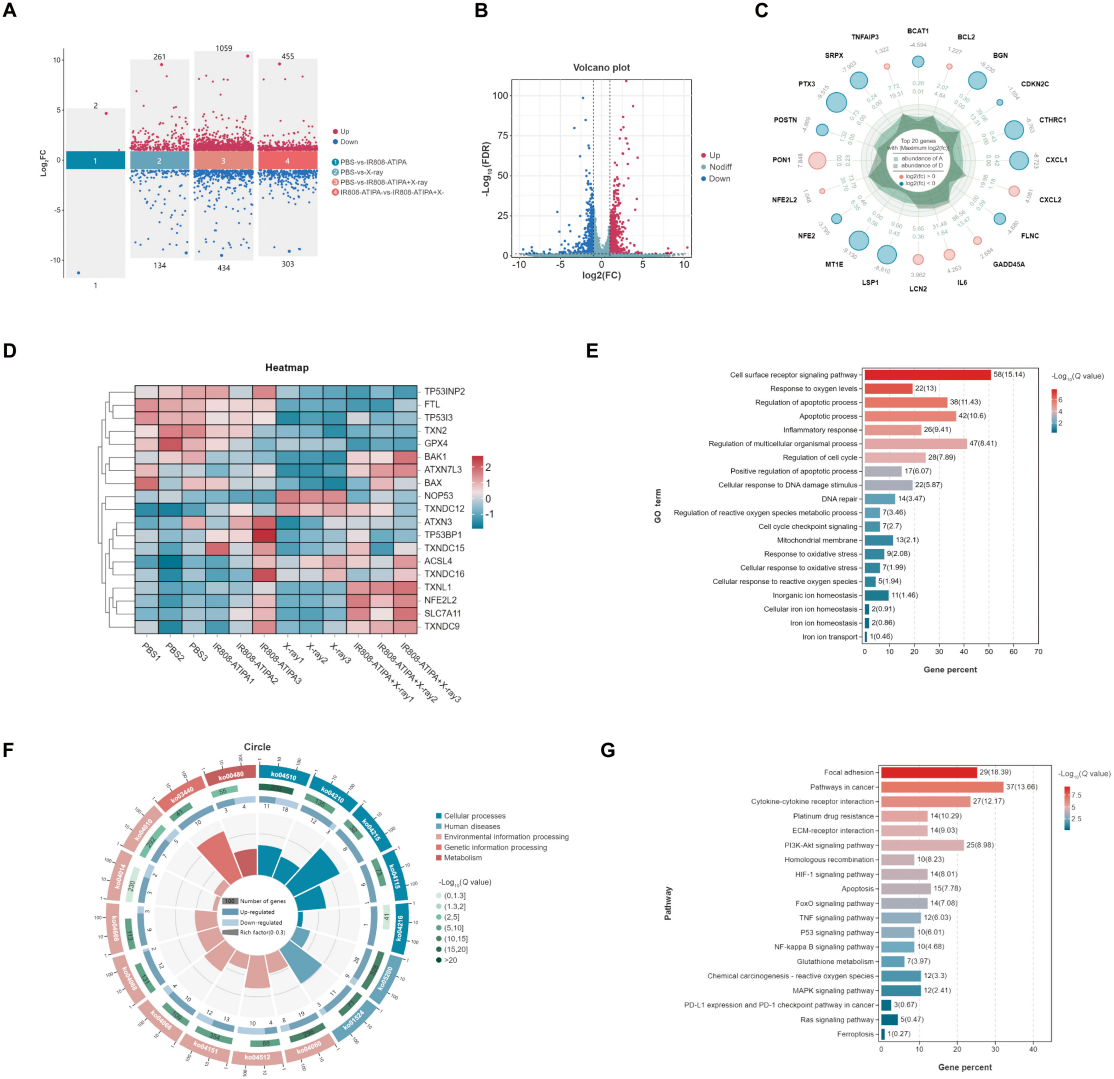
RNA-sequencing and DEG analysis in HeLa cells under various treatments. (A) Scatterplot showing the number of up- and down-regulated DEGs between groups. (B) Volcano plot comparing PBS group versus IR808-ATIPA + x-ray group. (C) Radar plot of the top 20 genes with the most significant differential expression between PBS and IR808-ATIPA + x-ray groups. (D) Bar chart of GO term enrichment for DEGs in the IR808-ATIPA + x-ray group. (E) Heatmap of DEGs related to apoptosis and ferroptosis across groups. (F) Circle plot of pathway enrichment. (G) Bar chart of KEGG pathway enrichment for DEGs in the IR808-ATIPA + x-ray group.

Significant differences were observed in the number of differentially expressed genes (DEGs) among the treatment groups (Fig. 3A). Compared to other groups, the IR808-ATIPA combined with x-ray irradiation group exhibited the largest number of DEGs relative to the PBS control (Fig. 3B). Analysis of the top 20 genes with the greatest differential expression between the PBS and IR808-ATIPA plus x-ray groups revealed that up-regulated genes were primarily associated with apoptosis, ferroptosis, and antioxidant responses. Conversely, down-regulated genes were mainly related to antioxidant defenses, inflammatory responses, and immune reactions (Fig. 3C). Heatmaps further illustrated clear distinctions in DEG expression patterns across groups, highlighting key genes involved in antioxidant defense, ferroptosis, and apoptosis pathways (Fig. 3D).

Gene Ontology (GO) enrichment analysis (Fig. 3E) showed significant enrichment of DEGs in biological processes such as “regulation of iron transport”, “cellular oxidative stress”, “apoptosis”, and “DNA damage repair” specifically within the IR808-ATIPA plus x-ray group. KEGG pathway analysis further confirmed significant enrichment in ferroptosis, apoptosis, iron metabolism, and oxidative stress-related pathways (Fig. 3F). Additionally, several critical cancer-related pathways, including the phosphatidylinositol 3-kinase (PI3K)-Akt, FoxO, mitogen-activated protein kinase (MAPK), and tumor necrosis factor (TNF) signaling pathways, were identified as being regulated by the combination of IR808-ATIPA and radiation (Fig. 3G).

### IR808-ATIPA induces ferroptosis damage in cells

**Fig. 4.**
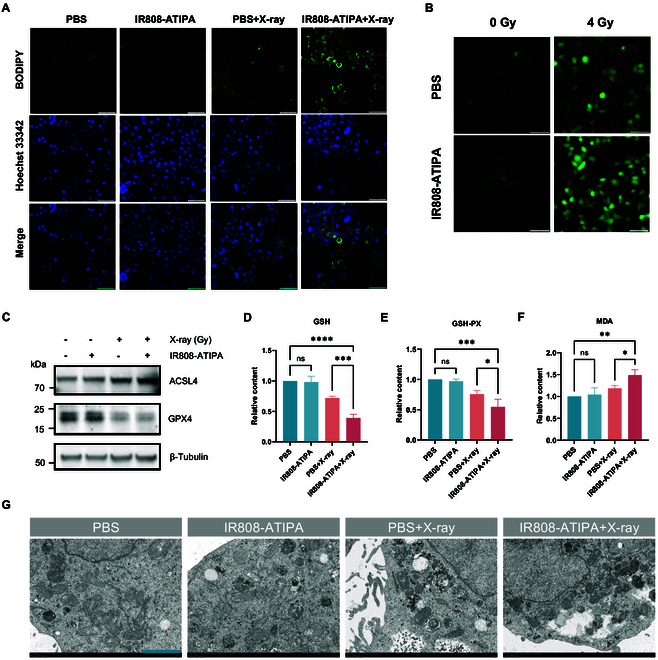
IR808-ATIPA treatment and x-ray irradiation induce DNA and morphological damage in HeLa cells. (A) BODIPY fluorescence images showing lipid droplet accumulation (scale bar, 200 μm). (B) Intracellular ROS levels detected by fluorescence (scale bar, 200 μm). (C) Quantification of intracellular GSH levels. (D) Measurement of intracellular GSH-PX levels. (E) Determination of intracellular MDA levels. (F) Western blot analysis of key proteins related to ferroptosis. (G) High-resolution transmission electron microscopy images of mitochondrial morphology (scale bar, 2 μm). Significance levels: ****P < 0.0001; ***P < 0.001; **P < 0.01; *P < 0.05; ns, not significant (*P* > 0.05).

Based on the RNA sequencing results, we validated whether IR808-ATIPA enhances sensitivity to ferroptosis during radiotherapy. After treating HeLa cells with IR808-ATIPA for 4 h followed by radiation exposure, significant morphological changes were observed, characterized by an increased number of intracellular lipid droplets, consistent with previous reports (Fig. 4A). LPO directly results in increased production of ROS. Fluorescence staining with the DCFH-DA probe (Fig. 4B) demonstrated a substantial elevation in intracellular ROS levels upon x-ray irradiation alone; importantly, ROS levels were further increased by the combined IR808-ATIPA and radiation treatment. These results indicate that IR808-ATIPA effectively amplifies radiation-induced ROS generation.

Radiation treatment reduced intracellular antioxidant markers such as GSH and GSH-PX levels, whereas MDA, a product of LPO, increased. The addition of IR808-ATIPA markedly accentuated these differences between treatment groups (Fig. 4C to E). IR808-ATIPA treatment alone, however, did not significantly alter the levels of GSH, GSH-PX, or MDA compared to controls. Ferroptosis-related protein expression analysis (Fig. 4F) revealed a pronounced decrease in GPX4 levels with combined IR808-ATIPA and radiation treatment compared to radiation alone. ACSL4 expression levels were also increased by the combination treatment, aligning closely with the transcriptomic data.

Changes in mitochondrial morphology provided further evidence supporting ferroptosis induction (Fig. 4G). IR808-ATIPA treatment alone minimally impacted mitochondrial morphology. Cells exposed to radiation alone (4 Gy) exhibited slight mitochondrial shrinkage. In contrast, cells pre-incubated with IR808-ATIPA for 4 h before radiation showed distinct ferroptosis-associated mitochondrial changes after 72 h, including reduced mitochondrial volume, rounded shape, enhanced electron density, ruptured mitochondrial membranes, increased outer membrane density, and significantly fewer mitochondrial cristae.

### CT imaging capabilities of IR808-ATIPA

**Fig. 5.**
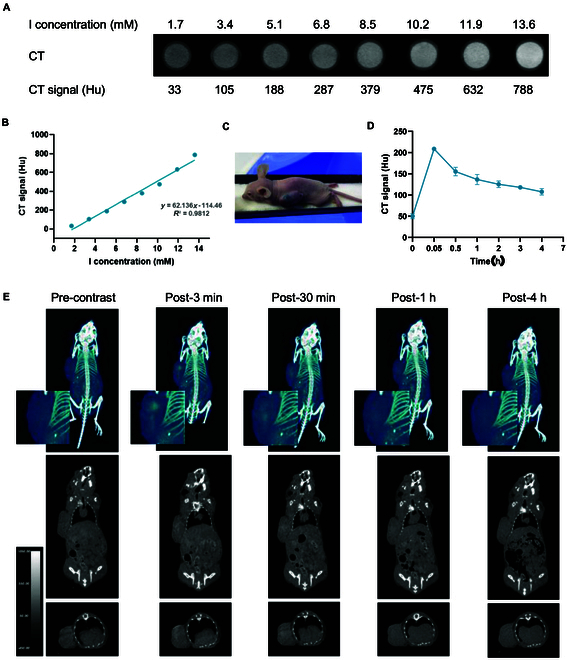
CT imaging performance of IR808-ATIPA. (A and B) CT values and signal curve for varying concentrations of IR808-ATIPA. (C) Image of drug-injected tumor in a nude mouse. (D) Time-dependent CT value changes in nude mice after intratumoral injection of IR808-ATIPA. (E) CT images at different time points post-injection of IR808-ATIPA in HeLa tumor-bearing nude mice. Data are presented as mean ± SEM. Significance levels: ****P < 0.0001; ***P < 0.001; **P < 0.01; *P < 0.05; ns, not significant (*P* > 0.05).

Due to its high iodine content, IR808-ATIPA demonstrated substantial potential as a CT imaging contrast agent. In vitro experiments showed a linear relationship between IR808-ATIPA concentration and corresponding CT values, confirming its excellent contrast properties (Fig. 5A and B).

In vivo, following intratumoral injection in nude mice, IR808-ATIPA rapidly accumulated within tumors, resulting in an immediate increase in CT signal intensity. These CT values peaked shortly after injection and remained significantly elevated for up to 4 h post-injection, demonstrating favorable retention and sustained enhancement for precise tumor delineation, essential for radiotherapy planning (Fig. 5C to E). As a comparison, intratumoral injection of iohexol at equivalent iodine concentrations resulted in rapid CT signal decay, with values returning to baseline levels approximately 2 h post-injection (Fig. S2).

### In vivo radiosensitization effect of IR808-ATIPA

**Fig. 6.**
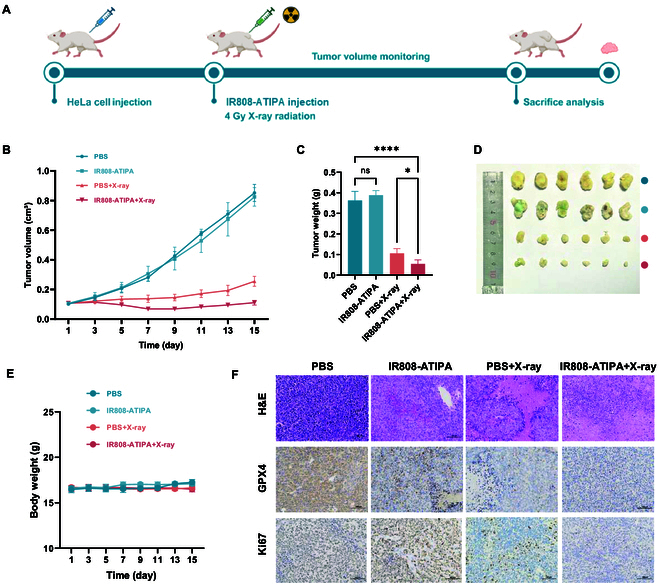
Efficacy of IR808-ATIPA in in vivo radiotherapy. (A) Timeline of treatment. (B) Tumor growth curves during treatment. (C) Tumor weights at dissection. (D) Photographs of tumors at the end of treatment. (E) Body weight curves of mice during treatment. (F) H&E, GPX4, and Ki67 staining images of tumor tissues at treatment endpoint (scale bar, 200 μm). Data are presented as mean ± SD (*n* = 6). Significance levels: ****P < 0.0001; ***P < 0.001; **P < 0.01; *P < 0.05; ns, not significant (*P* > 0.05).

We evaluated IR808-ATIPA’s enhancement of radiotherapy in vivo using a HeLa xenograft model. After injecting saline or IR808-ATIPA into subcutaneous tumors followed by radiotherapy, tumor growth was significantly inhibited in the IR808-ATIPA plus radiation group (Fig. 6A). Tumor weight data showed almost complete suppression in the IR808-ATIPA-treated group after 15 d (Fig. 6B to D). No significant weight loss was observed in treated mice, indicating good biocompatibility (Fig. 6E). Pathological analysis revealed extensive cellular damage and signs of ferroptosis in the combined treatment group, with decreased Ki67 levels indicative of reduced proliferation (Fig. 6F). Histological analysis further confirmed that IR808-ATIPA enhanced radiotherapy effects, showing tissue disorganization and necrosis consistent with ferroptosis. Immunohistochemistry demonstrated down-regulation of Ki67 and GPX4, aligning with RNA sequencing results.

### Biocompatibility assessment

**Fig. 7.**
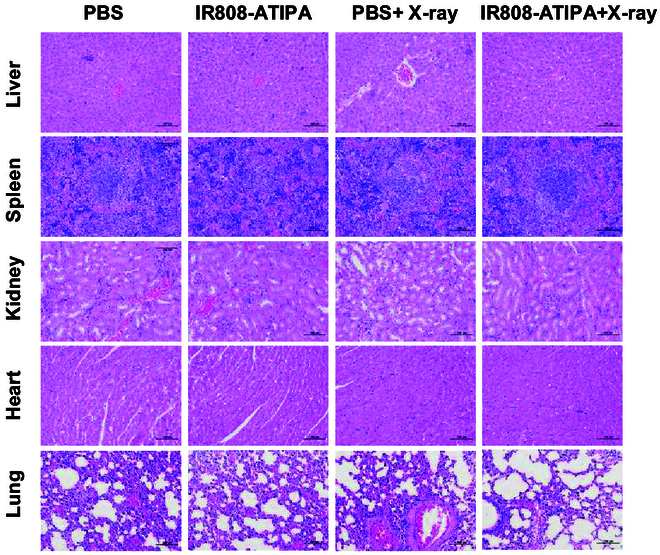
Biocompatibility assessment of IR808-ATIPA in vivo. Histological analysis of major organs post-treatment using H&E staining (scale bar, 200 μm).

The biocompatibility of IR808-ATIPA was assessed through H&E staining of major organs, which showed no significant damage or metastasis (Fig. 7A). Blood analysis for markers like AST, ALT, ALP (alkaline phosphatase), TIBL (total bilirubin), UREA, and CREA (creatinine) indicated no adverse effects on organ function (Table S3), suggesting minimal systemic toxicity and supporting IR808-ATIPA’s potential for CT-guided cancer therapy.

## Discussion

Although radiotherapy is widely used in clinical tumor treatment, its efficacy is limited by side effects on normal tissues, and precise radiotherapy requires real-time imaging guidance to overcome this challenge. By rationally designing the molecular structure of IR808-ATIPA, we successfully integrated the dual functions of CT imaging guidance and radiosensitization.

This innovative approach breaks the traditional separation of diagnosis and treatment, offering new insights for developing small-molecule theranostic drugs. Clinically used iodine-based contrast agents like iohexol and iodixanol rely on the core structure of ATIPA, but their small-molecule nature results in a short imaging window. IR808, an efficient near-infrared dye with excellent tumor-targeting capabilities, is widely used in the conjugation of anticancer drugs [31–33]. Thus, we covalently linked IR808 with ATIPA to create IR808-ATIPA, marking the first instance of combining a radiosensitizer with a cyanine dye. The larger molecular weight of IR808-ATIPA enables prolonged imaging, facilitating precise delineation of lesion target areas before radiotherapy and enhancing subsequent therapeutic effects. This optimization of molecular size likely extends retention time by enhancing the enhanced permeability and retention (EPR) effect in tumor tissue, overcoming the short imaging window caused by the rapid metabolism of small-molecule iodine agents.

It is worth noting that while metal-based radiosensitizers exhibit significant sensitization effects, they face challenges in clinical applications, such as long metabolic cycles of certain metal materials, which may lead to chronic toxicity due to accumulation in the body [34], and hydroxyl radicals (·OH) generated by metal nanoparticles via the Fenton reaction, which could cause oxidative damage to normal tissues [35]. In contrast, IR808-ATIPA, as a small-molecule iodine compound, activates the ferroptosis pathway in tumor cells, with its LPO regulated by GPX4 negative feedback, demonstrating a superior therapeutic window.

In vitro experiments confirmed the low toxicity of IR808-ATIPA and its radiosensitizing effect in radiotherapy. When IR808-ATIPA was added, the damage to cells caused by radiotherapy increased, inducing more apoptosis.

Ferroptosis is a non-apoptotic, regulated form of cell death triggered by LPO and dependent on iron and ROS [36–38]. Numerous studies indicate that ferroptosis is closely linked to cell death and tumor suppression induced by radiotherapy [39–42]. Consistent with this, our RNA sequencing analysis revealed that the combination treatment of IR808-ATIPA with x-ray irradiation significantly up-regulated pro-apoptotic genes, including BCL2 and GADD45A, and ferroptosis-associated markers such as LCN2. Concurrently, the expression of key antioxidant genes, including MT1E and NFE2, was notably suppressed. These findings suggest that IR808-ATIPA enhances radiotherapy efficacy by overcoming tumor resistance through a dual mechanism: It up-regulates BCL2 to counteract the anti-apoptotic effects mediated by Bcl-xL, while down-regulation of MT1E reduces the radical-scavenging capacity of metallothioneins, thereby amplifying oxidative damage in tumor cells. This dual-action approach offers a synergistic advantage, effectively addressing limitations associated with conventional radiosensitizers that typically rely on single-mechanism strategies. Furthermore, activation of hypoxia-inducible factor-1α (HIF-1α) observed in our results may reflect IR808-ATIPA’s regulatory effects on the tumor hypoxic microenvironment. Specifically, the induction of HIF-1α target genes such as BNIP3 could enhance mitochondrial autophagy, accelerating ferroptosis. Additionally, disruptions in the glutathione metabolic pathway observed in the treated group directly contribute to the loss of GPX4 enzymatic activity, a critical regulatory step in ferroptosis execution.

However, we observed compensatory activation of the NFE2L2 pathway, with NFE2L2 up-regulation in the IR808-ATIPA + x-ray treatment group. NFE2L2 up-regulation is associated with ferroptosis suppression, reducing ROS accumulation by increasing FSP1 expression [43] and inhibiting ferroptosis, while also decreasing ALOX12 activity by up-regulating SLC7A11, thus suppressing polyunsaturated fatty acid peroxidation and further inhibiting ferroptosis [44]. Nevertheless, our animal experiment data suggest that this adaptive response did not significantly undermine overall efficacy, possibly due to a “dual-pathway synergy” effect. The parallel activation of ferroptosis and apoptosis creates a dual mechanism, where even if one pathway is inhibited, the other can still sustain cell killing. The prolonged retention of IR808-ATIPA in tumor cells may overcome the transient protection mediated by NFE2L2 by extending oxidative stress exposure. Thus, we hypothesize that in this study’s radiotherapy, cells enhance their antioxidant capacity by up-regulating genes like NFE2L2 to cope with radiation-induced oxidative damage [45].

Following IR808-ATIPA combined with radiotherapy, gene expression changed significantly. The IR808-ATIPA + x-ray group showed clear enrichment in both ferroptosis and apoptosis pathways. Compared to previously reported single ferroptosis pathways, IR808-ATIPA combined with radiotherapy activates both pathways simultaneously [46]. We believe that this difference may stem from the specificity of the molecular design. This synergistic multimodal cell death mechanism holds significant clinical importance, enhancing radiotherapy sensitivity by disrupting cellular redox balance, compensating for the vulnerability of traditional apoptosis pathways to tumor cell evasion, and potentially breaching the protective mechanisms of the tumor microenvironment by combining x-ray-induced DNA damage with IR808-ATIPA-induced LPO.

Based on RNA sequencing results, we validated ferroptosis-related cellular damage, confirming that the damage aligns with ferroptosis characteristics, morphologically marked by small, deformed mitochondria with high-density membranes. IR808-ATIPA increased intracellular iron accumulation, which is linked to radical production, fatty acid supply, and LPO—key factors in inducing ferroptosis [38,47,48]. This process is induced by GPX4 reduction, and the down-regulation of GPX4 leads to LPO formation in cancer cells, suggesting that ferroptosis may be a critical pathway in IR808-ATIPA + x-ray combined therapy.

In animal experiments, we evaluated the antitumor efficacy of IR808-ATIPA under CT imaging guidance by monitoring tumor growth dynamics. The results demonstrated significant tumor-targeted accumulation and prolonged intratumoral retention of IR808-ATIPA in cervical cancer-bearing mice, with peak tumor CT enhancement observed at approximately 3 min post-injection and sustained imaging contrast lasting up to 4 h. Furthermore, combined treatment with IR808-ATIPA and x-ray irradiation markedly suppressed tumor growth in HeLa xenograft mouse models. Throughout the treatment period, no significant differences in survival rates or body weights were noted among different experimental groups. Endpoint histopathological examinations and comprehensive blood analyses further verified the biosafety and minimal systemic toxicity of IR808-ATIPA.

Additionally, IR808 itself possesses notable phototoxicity under near-infrared irradiation at an emission peak of 808 nm, demonstrating efficient tumor-targeting and near-infrared imaging capabilities. These properties indicate that IR808-ATIPA holds potential as an effective agent for fluorescence-guided surgery (FGS), particularly valuable for deeply located cervical cancers in the pelvic cavity. Future studies will explore dual-modality CT/fluorescence imaging to enable precise preoperative planning and real-time surgical navigation. Such an approach promises to facilitate multimodal imaging integration with phototherapy and radiotherapy, ultimately advancing the field toward comprehensive theranostic strategies in cancer treatment.

The integrated tumor radiotherapy and diagnostic model achieved in this study offers clear advantages. IR808-ATIPA enables immediate imaging post-injection, allowing dynamic adjustments to radiotherapy plans. Additionally, its tumor retention window of over 4 h covers the positioning and irradiation cycles of conventional fractionated radiotherapy, providing a new solution to target displacement issues in IGRT. However, this study has limitations, as the mechanistic research primarily focused on the HeLa cell line, while cervical cancer exhibits high heterogeneity. Future studies should validate the generalizability of these findings in primary cells.

The compound we synthesized, IR808-ATIPA, leverages its high iodine content and large molecular weight to provide excellent contrast in CT imaging, effectively aiding target delineation before radiotherapy. IR808-ATIPA not only enhances radiotherapy efficacy by increasing x-ray absorption but also heightens tumor cell sensitivity to radiation by inducing the ferroptosis pathway. Thus, IR808-ATIPA offers real-time guidance and monitoring for synergistic anticancer therapy, representing an innovative small-molecule strategy for integrated tumor diagnosis and treatment.

## Data Availability

The datasets used and/or analyzed during the current study are available from the corresponding author on reasonable request.
