# Supplementary material for: IR808-ATIPA: A Dual-Function Agent for Enhanced Computed Tomography Imaging and Radiotherapy Sensitization in Cervical Cancer Treatment
Source: Biomater Res. 2025 Aug 18;29:0222. doi: 10.34133/bmr.0222 (PMC12358750; doi:10.34133/bmr.0222)
Supplement: Supplementary 1 — Figs. S1 and S2 Tables S1 to S3 [file bmr.0222.f1.docx]

**SUPPLEMENTARY MATERIALS**


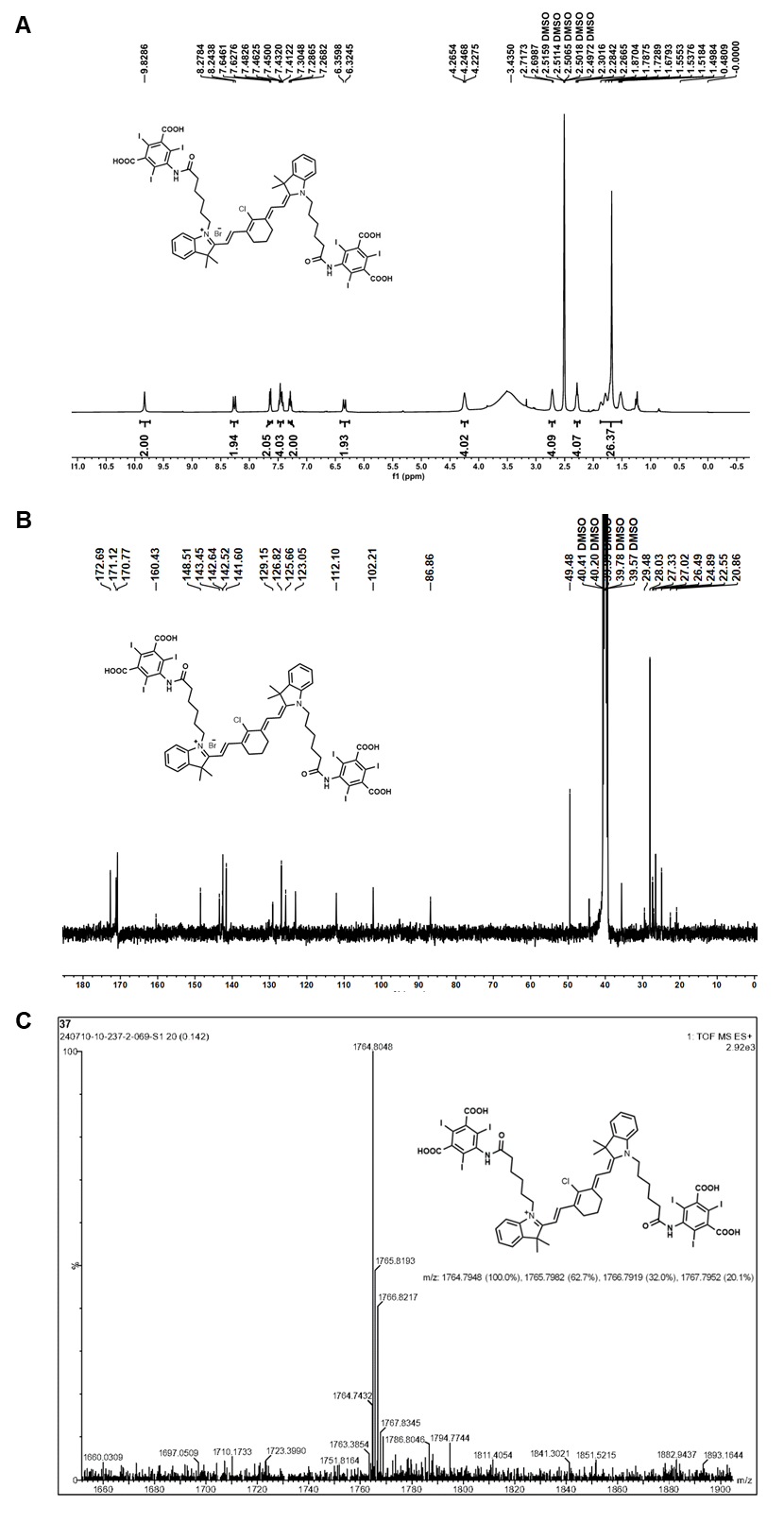


Figure S1. (A) High-resolution mass spectrum of IR808-ATIPA. (B) Proton NMR spectrum of IR808-ATIPA. Conditions: 400 MHz, DMSO-d6, δ (ppm), J (Hz). (C) Carbon NMR spectrum of IR808-ATIPA. Conditions: 101 MHz, DMSO-d6, δ (ppm).


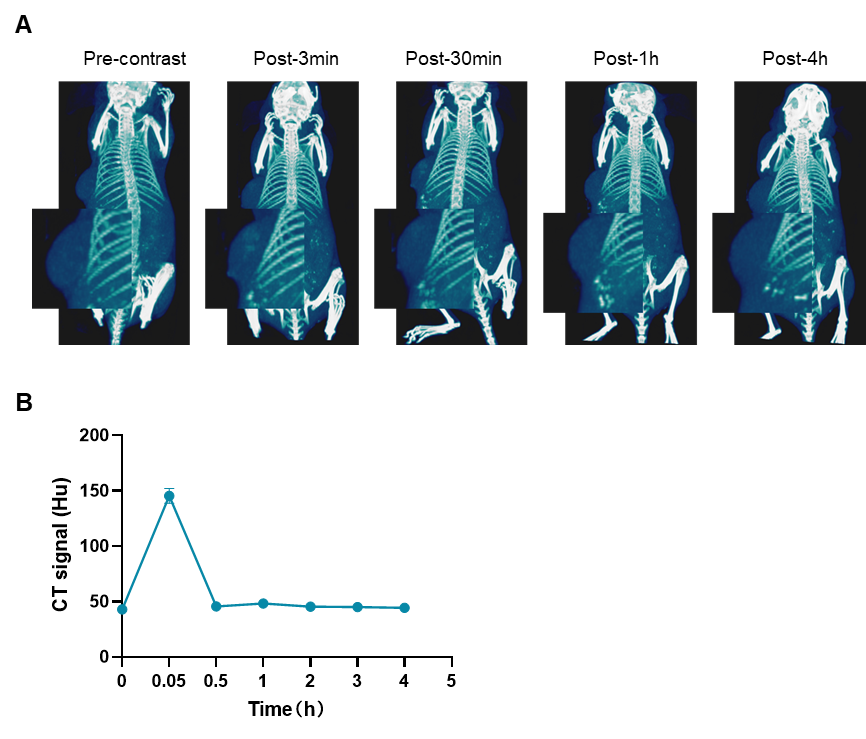


Figure S2. (A) Representative CT images at various time points after iohexol administration in HeLa tumor-bearing nude mice. (B) Time-dependent changes in CT values following intratumoral injection of iohexol in nude mice.

Table S1. ^1^H NMR (400 MHz, DMSO-d6)

| **Chemical Shift (δ)** | **Integration** | **Multiplicity (*J*, Hz)** | **Assignment** |
| --- | --- | --- | --- |
| 9.83 | 2H | s | Amide protons |
| 8.26 | 2H | d (*J*=13.9) | Methine protons |
| 7.64 | 2H | d (*J*=7.4) | Benzene ring protons |
| 7.50–7.40 | 4H | m | Benzene ring protons |
| 7.29 | 2H | t (*J*=7.3) | Benzene ring protons |
| 6.34 | 2H | d (*J*=14.1) | Methine protons |
| 4.24 | 4H | t (*J*=7.7) | Ortho-methylene protons to the amine group |
| 2.71 | 4H | t (*J*=7.4) | Cyclohexyl protons |
| 2.28 | 4H | t (*J*=7.0) | Protons adjacent to the carbonyl group |
| 1.87–1.508 | 26H | m | Terminal methyl protons/ Cyclohexyl protons/ Methylene linking groups |

Table S2. ^13^C NMR (101 MHz, DMSO-d6)

| **Chemical Shift (δ)** | **Carbon Type Assignment** |
| --- | --- |
| 172.69 | Carboxyl carbon |
| 171.12 | Amide carbon |
| 170.77 | Amide carbon |
| 148.51-102.21 | Aromatic/heterocyclic/alkene carbons |
| 86.86 | Alkyl carbon linking with quaternary ammonium salt |
| 49.48–20.86 | Alkyl carbons |

Table S3. Blood chemistry data after treatment.

| **Parameter** | **Value** | **Unit** | **Normal Range** |
| --- | --- | --- | --- |
| LT | 61.537 | U/L | 10.06-96.47 |
| AST | 196.070 | U/L | 36.31-235.48 |
| TBIL | 33.123 | μmol/l | 6.09-53.06 |
| DBIL | 9.977 | μmol/l | 0.45-33.98 |
| ALB | 28.294 | g/l | 21.22-39.15 |
| ALP | 86.480 | U/L | 22.42-474.35 |
| γ-GT | 0.575 | U/L | 0-7.78 |
| TBA | 4.262 | μmol/l | 0-8.51 |
| UREA | 12.553 | mmol/l | 10.81-34.74 |
| CREA | 49.570 | μmol/l | 10.91-85.96 |
| UA | 178.473 | μmol/l | 44.42-224.77 |
| CK | 1938.953 | U/L | 0-2970.55 |
| LDH1 | 23.928 | U/L | 0-37.07 |
